# Treating newly split *Apis mellifera* honey bee colonies with organic miticides—an opportunity for Integrated Pest Management of *Varroa destructor* mites (Mesostigmata: Varroidae)

**DOI:** 10.1093/jee/toaf126

**Published:** 2025-07-01

**Authors:** Dan Aurell, Selina Bruckner, Todd D Steury, Geoffrey R Williams

**Affiliations:** Bee Center, Department of Entomology and Plant Pathology, Auburn University, Auburn, AL 36849, United States; Bee Center, Department of Entomology and Plant Pathology, Auburn University, Auburn, AL 36849, United States; College of Forestry, Wildlife and Environment, Auburn University, Auburn, AL 36849, United States; Bee Center, Department of Entomology and Plant Pathology, Auburn University, Auburn, AL 36849, United States

**Keywords:** apiculture, invasive parasites, brood break / interruption, acaricides, agricultural IPM

## Abstract

Parasitism from *Varroa* mites (*Varroa destructor* Anderson and Trueman, Mesostigmata: Varroidae) is a major driver of honey bee colony losses *(Apis mellifera* L., Hymenoptera: Apidae). While synthetic miticides are valuable for *Varroa* management, high reliance on these compounds has selected for miticide-resistant *Varroa* populations. To enable more sustainable *Varroa* management and provide options when synthetic miticides such as amitraz fail, effective Integrated Pest Management (IPM) approaches are urgently needed. Here, we show that organic miticides (oxalic acid, “OA”; and hops beta acids) can achieve high efficacy against *Varroa* when strategically combined with a widely used cultural control (starting new colonies, “splits,” with queen cells). This common splitting practice acts as a cultural control by temporarily reducing the amount of brood (developing bees) in colonies. This forces *Varroa* mites out of the protected environment of brood cells and on to adult bees—where they can be more effectively targeted with miticides. Based on *Varroa* infestation rates of adult bees, we determined that the organic miticide treatments OA dribble (75.5% efficacy), 5× OA dribble (82.2%), and HopGuard (82.7%) were significantly more effective than no treatment and provided comparable efficacy to amitraz-based miticides. In contrast, OA vapor (44.3%) did not provide effective *Varroa* control. Based on observations of queen and colony success, colony strength measurements, and hive weights, none of the organic miticides showed signs of harming colonies. Therefore, this combination of cultural and chemical control provides an additional opportunity for beekeepers to implement IPM for more effective and sustainable *Varroa* management.

## Introduction

Damage from *Varroa* mites (*Varroa destructor* Anderson and Trueman, Mesostigmata: Varroidae) is considered the leading cause of mortality of honey bee colonies (*Apis mellifera* L., Hymenoptera: Apidae) in major beekeeping regions (Guzmán-Novoa et al. 2010, Steinhauer et al. 2018, Aurell et al. 2024a). These parasitic mites damage colonies by feeding on adult bees and brood (developing bees) and by acting as a vector of pathogenic viruses (Traynor et al. 2020). The issue has become exacerbated by the emergence of *Varroa* mites resistant to amitraz, the synthetic miticide that US beekeepers largely use to manage *Varroa* (Haber et al. 2019, Rinkevich 2020, Rinkevich et al. 2023). To manage resistant arthropods, an important strategy is to implement rotations between pesticides with different active ingredients (Whalon et al. 2008). Developing techniques to use miticides in rotation will enhance beekeepers’ ability to manage *Varroa* in ways that align with Integrated Pest Management (IPM) recommendations (Jack and Ellis 2021).

Miticides based on naturally occurring active ingredients (“organic miticides”) provide options for rotation in *Varroa* management programs. Products based on oxalic acid, hops beta acids, formic acid, and thymol are all EPA-registered and available in the United States (US EPA 2024). However, these miticides are not effective against *Varroa* in all conditions (Honey Bee Health Coalition 2022). Besides formic acid, these organic miticides are primarily effective against *Varroa* in the dispersal phase (on adult bees) but do not effectively pass through the wax capping of the brood cell to reach *Varroa* in the reproductive phase (on developing bees in capped brood cells) (eg Lupo and Gerling 1987, Gregorc and Planinc 2001). For example, short-acting oxalic acid applications can have over 99% efficacy against *Varroa* under broodless conditions, but only ~40% to 50% efficacy when brood is present (Gregorc and Planinc 2001). Similarly, greater efficacy has been obtained with beta hops acids in low-brood conditions than when brood is abundant (DeGrandi-Hoffman et al. 2014). For maximum efficacy, applications of oxalic acid and beta hops acids thus need to be strategically timed to coincide with brood breaks (periods when colonies contain little or no capped brood).

A common beekeeping management technique—splitting—can provide short brood breaks that can be exploited for *Varroa* management. When existing colonies (“parent colonies”) are split, one or more new queenless units (“splits”) are created (Connor 2014). When new colonies are provided with a queen cell (immature queen) or are allowed to raise their own queen, a brood break results. This is because the time needed for the immature queen to emerge, mate, and begin laying eggs (DeGrandi-Hoffman et al. 2014) allows most or all of the brood in the split to complete development and emerge as adult bees. This serves as a cultural control by depriving *Varroa* mites of the brood they need for reproduction, and forces them into the dispersal phase on adult bees where they are unprotected from treatments. Beekeepers can exploit brood breaks as an opportunity to use miticides with high efficacy (Lupo and Gerling 1987, Gregorc et al. 2017, Büchler et al. 2020). This study focuses on treatment of splits provided with queen cells, as this is a widespread management practice among large-scale beekeeping operations in the United States. If beekeepers make walk-away splits (splits that are allowed to raise their own queen), the colonies experience a longer brood break that can be used as a treatment opportunity, but if a mated queen is introduced, a brood break does not occur.

Previous research indicates that starting colonies with queen cells can be effectively combined with organic miticides. While not peer-reviewed, Oliver (2013) has performed the most comprehensive tests to date of organic miticide treatments in conjunction with splitting and installing a queen cell. These tests showed promising results with oxalic acid dribble applied 15 or 19 d after splitting (Oliver 2013). As well, hops beta acids applied 19 d after splitting achieved moderately effective *Varroa* control (Oliver 2013, DeGrandi-Hoffman et al. 2014), and in additional studies in broodless (Rademacher et al. 2015) and low-brood conditions (Kulhanek et al. 2023), hops beta acids have been effective. However, more information is needed to guide beekeepers’ choice of which miticides to use.

Open questions include the relative efficacy against *Varroa* of different organic miticide options, and their effects on newly established honey bee colonies. Two of the organic miticides (oxalic acid and hops beta acids) are considered safe for small colonies at warm temperatures: small hives (nucs) are mentioned on both labels, oxalic acid has no maximum application temperature, and HopGuard has a 38°C maximum application temperature (US EPA 2021a, 2021b). Oxalic acid can be applied using several application methods, justifying additional treatment groups. Oliver (2013) suggested possible improvements of the oxalic acid and hops beta acids treatments by applying the product not only during the low-brood period but also early in colony establishment. Additionally, the occurrence of a brood break could synchronize the cell invasion of large cohorts of *Varroa*, enabling targeting of *Varroa* mites at colony establishment, on day 18, and around day 41 when such cohorts of *Varroa* would be expected to exit brood cells after reproduction. Finally, it is important to compare the organic miticides against registered and unregistered amitraz-based treatments that are used in the beekeeping industry (Honey Bee Health Coalition 2021).

To fill these knowledge gaps, we investigated: (i) the efficacy against *Varroa* of miticides applied to new colonies that received queen cells; and (ii) whether miticides applied in this setting cause harm to queens, colonies, or colony productivity.

## Materials and Methods

We conducted the experiment in Auburn, AL, United States during the spring and summer of 2022. On day 0, we removed queens and divided strong colonies (“parent colonies”) in double deep brood chambers to make standardized experimental colonies (“splits”) in single brood chambers. We then moved splits to experimental apiaries and provided each split with an immature queen (queen cell). We applied miticides and assessed *Varroa* infestation, queen establishment, adult bee population, and colony weight, as described below. We have summarized the timing of actions, assessments, and treatments in an experiment timeline (Supplementary Fig. S1).

### Colony Establishment and Management

We made 4 cohorts of splits between 8 and 25 March 2022. Each cohort (those splits made on the same date) was moved to its own experimental apiary, totaling 177 experimental colonies in 4 apiaries, with 35 to 50 splits per cohort. On day 0 (the day of splitting), we removed queens from parent colonies and rearranged resources so that every brood chamber (future split) contained 3 frames of brood, 2 frames of honey, 3 frames of foundation, and one 5.7-L frame (1.5 gallon) feeder, in one 10-frame deep-size Langstroth brood chamber (interior volume: 42.5 L). We removed extra frames containing brood or other resources. We made 2 to 5 splits from each parent colony depending on its strength and stacked the splits in the original hive location to allow bees to distribute overnight, with splits having ~3 to 5 frames of bees. On the morning of day 1, we separated the splits and moved them to an experimental apiary >3 km away. On day 2, we installed queen cells in cell protectors (10 d after grafting), applied miticides if relevant, and fed 5.7 L of 1:1 (w/w) sugar syrup. For each cohort, all queen cells were grafted from the same breeder queen.

We continued springtime management: checking queen status, feeding, and adding boxes. On day 30, we fed 5.7 L of syrup and added a second deep brood chamber to each colony. On day 51, we fed 5.7 L of syrup to the first 3 cohorts, but not in the last cohort as colonies were storing sufficient nectar. We added a first honey super on days 58 to 59 to all colonies and added additional supers as needed on an individual-colony basis, when colonies had more than half of their top super filled with honey.

Some colonies were removed from the trial entirely or had their data excluded from analyses (see “Statistics” section). On days 44 and 58, we noted visual signs of European foulbrood (EFB) in a total of 19 colonies, quite evenly across treatment groups. We removed these colonies from the trial to minimize the spread of disease. Queen issues by day 77 were as follows: queens did not establish (23 colonies), queenlessness (9), drone laying queens (4), and excessive weakness (5) (Supplementary Fig. S3).

### Miticide Treatments

Treatment assignments were performed separately for each of the 4 cohorts using the following stratified randomization procedure. To homogenize starting *Varroa* infestations between treatment groups, we stratified colonies (within cohort) by *Varroa* infestation of adult bees in parent colonies (0 to 38 *Varroa* in alcohol wash samples of ~300 bees; mean = 5.5) taken within 1 wk before splitting. Then, we randomly assigned treatments within strata, starting with the stratum with the highest *Varroa* infestation. All treatments were represented in each apiary and were spatially interspersed within the apiaries (Hurlbert 1984). Miticide treatment groups were as follows: (i) Control; (ii) Apivar; (iii) Amitraz EC; (iv) OA dribble; (v) 5× OA dribble; (vi) OA vapor; and (vi) HopGuard.

Control colonies underwent the cultural control of splitting with a queen cell but received no miticides. Except for Amitraz EC, we timed treatments to coincide with the period of low brood on approximately day 18 (Table 1). For single short-acting treatments (OA dribble, OA vapor), this meant applications on day 18. For the repeated OA dribble treatment, we treated on day 2 to target mites in the dispersal phase at time of queen cell placement, we treated on day 18 to coincide with a low-brood period, and then on days 35, 41, and 47 to coincide with a predicted range (depending on timing of queen egg-laying initiation) of days when *Varroa* mites were predicted to be exiting brood based on the hypothesis that the brood break would synchronize their entry into the earliest cohort of new appropriate-age brood cells. For the HopGuard treatment, we targeted the initial dispersal *Varroa* by applying it on day 2 as well as the period of low brood by reapplying on day 18. For Apivar, a long-acting treatment, we applied it on day 2 for efficiency. For Amitraz EC, we timed the application to coincide with checking queen status on day 30, which anecdotally is a common time to apply this treatment. We used amitraz-based treatments Amitraz EC and Apivar as positive controls. Apivar colonies received one strip of Apivar (amitraz 3.3%, polymer strip, Veto-Pharma, Palaiseau, France) as appropriate to the maximum colony strength of ~5 frames of bees (US EPA 2021c). Amitraz EC colonies received one half shop towel (14.6 × 27.0 cm; Scott) in between the brood chambers. This towel contained 12 mL of a 1:4 mixture by volume of amitraz EC concentrate (amitraz 12.5%, emulsifiable concentrate) and canola oil, for a mixture containing 2.5% amitraz (Aurell et al. 2024b). We prepared oxalic acid syrup following Sammataro et al. (2008). Briefly, we prepared OA sugar syrup using 75 g Api-Bioxal (oxalic acid dihydrate 100%, Chemicals Laif, Vigonza, Padua, Italy), 1 L hot water, and 1 kg white sugar for a batch volume of 1,700 mL. This 4.4% w/v oxalic acid dihydrate solution would conventionally be considered a 3.2% w/v oxalic acid solution after converting to the equivalent mass of pure oxalic acid (Sammataro et al. 2008, Oliver 2025). OA dribble colonies received 5 mL of OA sugar syrup per frame of bees (usually 15 to 25 mL). The 5× OA dribble colonies received oxalic acid sugar syrup as described above but received 5 sequential applications (Table 1). OA vapor colonies received 1 g of Api-Bioxal (US EPA 2021a) by heat vaporization using a ProVap 110 vaporizer (OxaVap, Manning, SC, USA) set to 230°C following manufacturer’s instructions. We applied oxalic acid vapor through a hole in the back corner of the hives and kept entrances closed for 10 min. HopGuard colonies received one strip of HopGuard 3 (potassium salt of hop beta acids 16%, BetaTec Hop Products, Washington, DC, USA) on 2 occasions (US EPA 2021b). At the end of the treatment period (on day 32), we removed both strips. We applied the strip treatments (Apivar and first strip of HopGuard) within 10 cm of the queen cell to assess possible negative effects on queens when strips were placed close to the cell.

**Table 1.** Miticide treatments tested to manage *Varroa destructor* infestations in newly established *Apis mellifera* colonies that were provided with queen cells (immature queens). Day 0 was defined as the date that the experimental units, “splits,” were prepared (including removal of the original queen). Queen cells were provided to the splits on day 2

Treatment group	Day(s) applied, [day removed]	Product, application and dosage per occasion	References
Control	N/A	N/A	N/A
Apivar	2, [44]	1 Apivar strip	(US EPA 2021c)
Amitraz EC	30, [51]	½ towel with 12 mL of 2.5% amitraz oil mix	(Aurell, Wall, et al. 2024)
OA dribble	18	3.2% Api-Bioxal sugar solution; 5 mL per frame of bees (up to 50 mL)	(Sammataro et al. 2008)
5× OA dribble	2, 18, 37, 41, 47	3.2% Api-Bioxal sugar solution; 5 mL per frame of bees (up to 50 mL)	(Sammataro et al. 2008)
OA vapor	18	1 g Api-Bioxal	(US EPA 2021a)
HopGuard	2,18, [32]	1 HopGuard strip (2 strips total)	(US EPA 2021b)

### Assessments—*Varroa*

To quantify *Varroa* infestations, we used an alcohol wash method (Dietemann et al. 2013). We used data from *Varroa* assessments on days 2 and 77, and on the day of honey harvest for analyses. To assess *Varroa* infestation rates, we shook bees from one or more frames containing older open brood, waited ~10 s for bees to fly away, and then collected one 118 mL (½ cup) sample of bees into a jar of 35% isopropyl alcohol. We added isopropyl alcohol solution until the jar contained at least 500 mL and shook it for 1 min. Then, we used sieves to separate *Varroa* from bees, rinsing the sample 3 times (Aurell et al. 2024b). For all data used in analyses, we counted both *Varroa* and bees in each sample. Day 2 samples contained a mean of 310.1 bees (min = 235; max = 422), day 77 samples contained a mean of 362.3 bees (min = 227; max = 516), and samples at honey harvest contained a mean of 340.4 bees (min = 237; max = 476). Treatment assignments were made based on assessments of parent colonies’ *Varroa* infestation rates <1 wk before splitting. For these samples, *Varroa* mites were counted but not bees. For context, we conducted field tests for amitraz resistance according to Rinkevich (2020) on 49 colonies on day 2.

### Assessments—Colony Condition

We assessed metrics of colony condition including queen status, queen establishment success, colony strength, and hive weight. To record issues with queens, we assessed queen status on days 18, 30, 44, 58, 72, 77, and at honey harvest (June 30 and July 1; days 97 to 115 depending on the cohort). Colonies were considered queenright when eggs were seen or a mated queen was seen. If we observed queenless or drone layer colonies, we recorded this. Queens were considered to have established if they successfully started laying worker brood. If we did not see eggs on day 30, the queen was considered not to have successfully established and we removed the colony from the experiment. To estimate the date when each queen began laying, we estimated the age of the oldest brood on days 18 and 30 (Human et al. 2013) and from this estimated the day of egg-laying initiation. On day 77, we assessed colony strength by visually estimating the percent coverage of bees on both sides of each frame, corrected for frame depth (a medium frame having 0.64 times the area of a deep frame), and then summarized this as “frames of bees” for the colony, in which 1 frame of bees represents a deep frame completely occupied on both sides with bees (Delaplane et al. 2013). At honey harvest, we weighed each hive (including supers). We corrected the hive weight to correspond to a hive with 2 brood chambers and 1 super by subtracting 5.4 kg for each additional super (ie the average weight of empty supers). We used hive weight as a proxy for honey production as honey is the heaviest resource in a honey bee nest (Seeley 1985).

### Statistics

For analyses of day 77 data (*Varroa* infestation, colony strength), we excluded a total of 60 colonies which had any issue by that date (Supplementary Fig. S3): queens did not establish, queenlessness, drone laying queens, and visual signs of EFB. Five colonies that were removed due to excessive weakness were excluded in analyses of *Varroa* infestation, but for colony strength, we included these colonies and to be conservative assigned them a strength of 0 frames of bees. For analyses of data from the time of honey harvest (*Varroa* infestation, hive weight), all the above colonies were excluded, plus an additional 10 colonies which had issues between day 77 and honey harvest. Issues were relatively evenly distributed between treatment groups (Supplementary Fig. S3). As a measure of “treatment success” based on directly measured data, we determined the percentage of colonies that had a *Varroa* infestation below 3% on day 77.

We performed data analysis in R version 4.2.2 (R Core Team 2022) relying on the *tidyverse* package (Wickham et al. 2019) for data manipulation and the statistical packages *MASS*, *lme4*, and *emmeans* (Venables et al. 2002, Bates et al. 2015, Lenth 2020). We used *ggplot2* for plotting (Wickham 2016) and used *gt* for table generation (Iannone et al. 2022). We fitted generalized linear models—with negative binomial distributed errors (*Varroa* infestation rate), binomial distributed errors (queen establishment success), or Gaussian distributed errors (colony strength and hive weight). Hive weight was modeled with a linear mixed model that included a random effect of apiary to account for the different periods between splitting and honey harvest. Model formulas are shown in Supplementary Table S1. We assessed the significance of individual predictors by stepwise deletion (Crawley 2013), using an alpha level of 0.05.

Although at the start of the trial, *Varroa* infestation rates did not differ significantly between treatments (χ^2^_6_ = 4.02; *P* = 0.674), we statistically controlled for pretreatment *Varroa* infestation in an ANCOVA framework (O’Connell et al. 2017). We fitted 1 model of day 77 *Varroa* infestation, and another model for *Varroa* infestation at honey harvest, both of which included day 2 *Varroa* infestation as a covariate (Supplementary Table S1). The model for *Varroa* infestation at honey harvest also included a covariate for the days elapsed between splitting and honey harvest—to account for the differing time spans between splitting and honey harvest in the four apiaries resulting from the staggered start. This also allowed us to normalize predictions to day 105 (the mean honey harvest date for the four apiaries). In models for the number of *Varroa* in samples of bees, we also included an offset term to account for the differing number of bees in different alcohol wash samples. Predictions of *Varroa* infestation were based on a day 2 *Varroa* infestation of 1.42%, reflecting the mean among colonies that were included in the analysis of *Varroa* infestations on day 77.

To validate this analysis, we also conducted a before-after-control-impact (BACI) analysis of *Varroa* infestation on days 2 and 77, considering the hive as the replicate (Smith 2002). Using a generalized linear mixed model (no. 6, Supplementary Table S1), we tested for the significance of the *Treatment* × *TimePoint* interaction and to test whether the change over time of *Varroa* infestations differed between treatments we performed a Tukey-adjusted contrast-of-contrasts procedure with the *emmeans* package.

To estimate efficacy of miticides against *Varroa*, we used a modification of the Henderson–Tilton equation (Henderson and Tilton 1955), which is used to compare posttreatment differences in pest abundance while correcting for pretreatment differences in pest abundance. This formula, Eq. (1), uses the count of pests in the treated condition before (*Tb*), control before (*Cb)*, treated after (*Ta)*, and control after (*Ca)*, to calculate efficacy.


$$\begin{array}{l} Ef\;\;f\;\;icacy\;\left(\%\right)=100\times \left(1-\frac{Ta\;\times \;Cb}{Tb\;\times \;Ca}\right) \end{array}$$ (1)

Calculating efficacy directly per colony with the Henderson–Tilton formula would not allow us to estimate uncertainty in terms of 95% confidence intervals, so we instead incorporated this efficacy calculation into our statistical modeling. Our models were of the form *VarroaAfter ~ VarroaBefore + Treatment* (Supplementary Table S1), which controlled for the pretreatment *Varroa* infestation and allowed us to simplify the Henderson–Tilton formula. As predictions from these models were based on all groups having the same starting infestation, *Cb* is equal to *Tb*, and the formula can be simplified to Eq. (2) to derive efficacy estimates from model predictions.


$$\begin{array}{l} Ef\;\;f\;\;icacy\;\left(\%\right)=100\times \left(1-\frac{Ta}{Ca}\right) \end{array}$$ (2)

The statistical models we fitted were used to estimate the ratio of *Ta*/*Ca* and confidence intervals around this estimate (using the *emmeans* package). We used Eq. (2) to transform point estimates and confidence limits of the *Ta*/*Ca* ratio to percent efficacy. In this formulation, the efficacy of the untreated control is considered 0, and to test whether treatment efficacy differs from 0, we tested whether *Ta*/*Ca* was significantly different from 1. A negative efficacy corresponds to higher *Varroa* infestations in a treated group than in the control group, and when the 95% confidence interval contains an efficacy of 0, this corresponds to a non-significant *P*-value for the efficacy differing from 0.

We estimated systematically lower treatment efficacies against *Varroa* at harvest than on day 77 (Supplementary Table S4), potentially indicating transmission of *Varroa* mites between colonies during the trial (Peck and Seeley 2019). Therefore, we focused on differences in *Varroa* infestation between treatments on day 77 and efficacies on day 77 as these likely represent more accurate estimates.

## Results

### *Varroa* Mites

Amitraz resistance testing on a subset of splits (*n* = 49) indicated 35.3% Apivar resistance of *Varroa* mites in the average split before treatment.

**Fig. 1.**
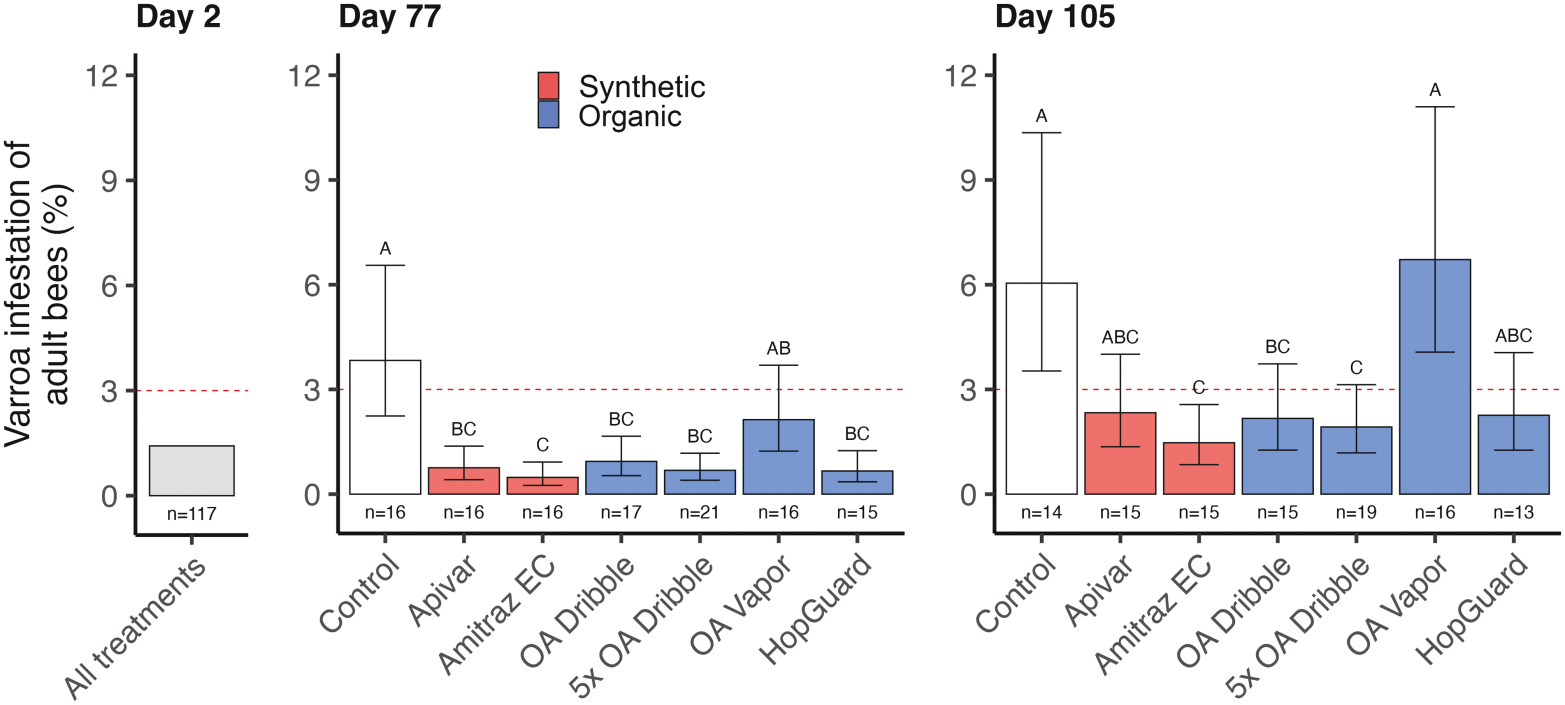
Effect of organic and synthetic miticides on *Varroa destructor* infestation rates in new *Apis mellifera* honey bee colonies started with queen cells (immature queens). Bars show *Varroa* infestation of adult bees on days 2, 77, and 105. Point estimates and 95% confidence intervals from fitted models are shown. Within each day, groups that do not share a letter are significantly different at *P* < 0.05. See Supplementary Table S2 for point estimates and confidence intervals; see Supplementary Figure S2 for directly plotted data. The horizontal dashed line indicates 3% *Varroa* infestation, above which treatment is commonly recommended.

Treatments significantly influenced *Varroa* infestation rates on day 77 (χ^2^ = 37.3; df = 6; *P *< 0.001), and at harvest (χ^2^ = 26.7; df = 6; *P *< 0.001). On day 77, all treatments except OA vapor provided lower *Varroa* infestations than the untreated Controls (Fig. 1; Supplementary Tables S2 and S3). Apivar resulted in significantly lower *Varroa* infestations than untreated Controls (*z* = −3.95; *P* = 0.002), as did Amitraz EC (*z* = −4.83; *P* < 0.001), OA dribble (*z* = −3.52; *P *= 0.008), 5× OA dribble (*z* = −4.44; *P *< 0.001), and HopGuard (*z* = −4.15; *P *< 0.001). The difference between Control and OA vapor was not significant (*z* = −1.49; *P *= 0.748). OA vapor had a higher *Varroa* infestation rate than Amitraz EC (*z* = 3.44; *P *= 0.011) (Supplementary Table S3).

**Fig. 2.**
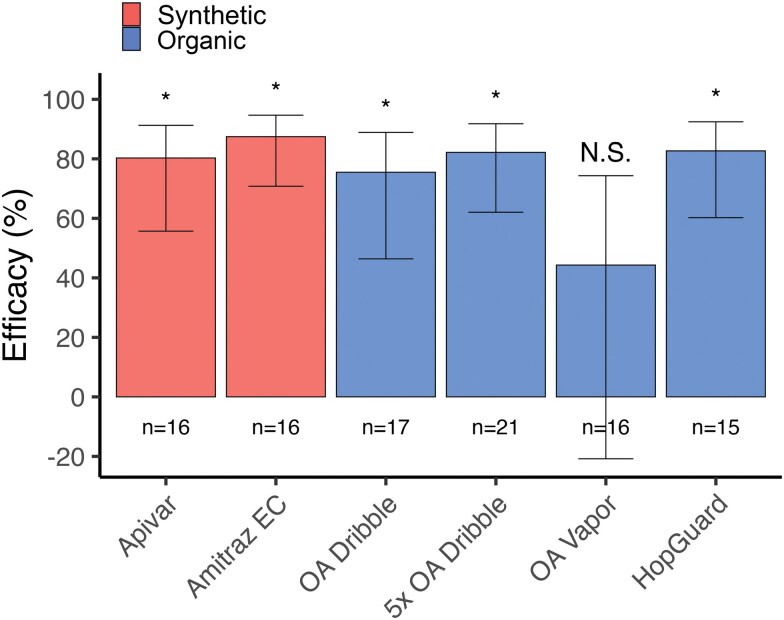
Efficacy of organic and synthetic miticides against *Varroa destructor* in *Apis mellifera* colonies started with queen cells (immature queens). Bars show the calculated efficacy of miticides in reducing *Varroa* infestation rates on day 77, and error bars show 95% confidence intervals. An asterisk indicates significantly efficacy against *Varroa* relative to untreated control colonies, *P* < 0.05. OA = Oxalic Acid.

We estimated miticide efficacies miticides based on the modeled differences in *Varroa* infestation rates on day 77 and calculated the percentage of colonies whose *Varroa* infestations were successfully kept below 3% based on measured *Varroa* infestations on day 77. From highest to lowest efficacy, these results are as follows: Amitraz EC (87.5% efficacy, 100% success); HopGuard (82.7% efficacy, 100% success), 5× OA dribble (82.2% efficacy, 100% success), Apivar (80.3% efficacy, 93.8% success), OA dribble (75.5% efficacy, 88.2% success), and OA vapor (44.3% efficacy, 62.5% success) (Fig. 2; Supplementary Table S4). Several of the estimated efficacies for organic miticides compared favorably to those of synthetic miticides. Estimated efficacies on day 77 and at harvest, along with confidence intervals, are also provided in Supplementary Table S4.

Correction for multiple comparison did not drive the lack of other significant differences between treatments, except that OA vapor would be considered inferior to all other treatments if not correcting for multiple comparison. Comparing treatment effects using the BACI procedure gave results that were qualitatively similar to the above comparisons based on ANCOVA modeling. As expected, the *Treatment* × *TimePoint* interaction was significant (χ^2^ = 27.8; df = 6, *P* < 0.001), and only one conclusion on the comparison of treatments differed: when compared using the BACI procedure the effects of OA vapor and Amitraz EC on *Varroa* infestation were not significantly different (*z*-ratio = 2.40; df = ∞; *P* = 0.201).

### Treatment Effects on Honey Bee Colonies

**Fig. 3.**
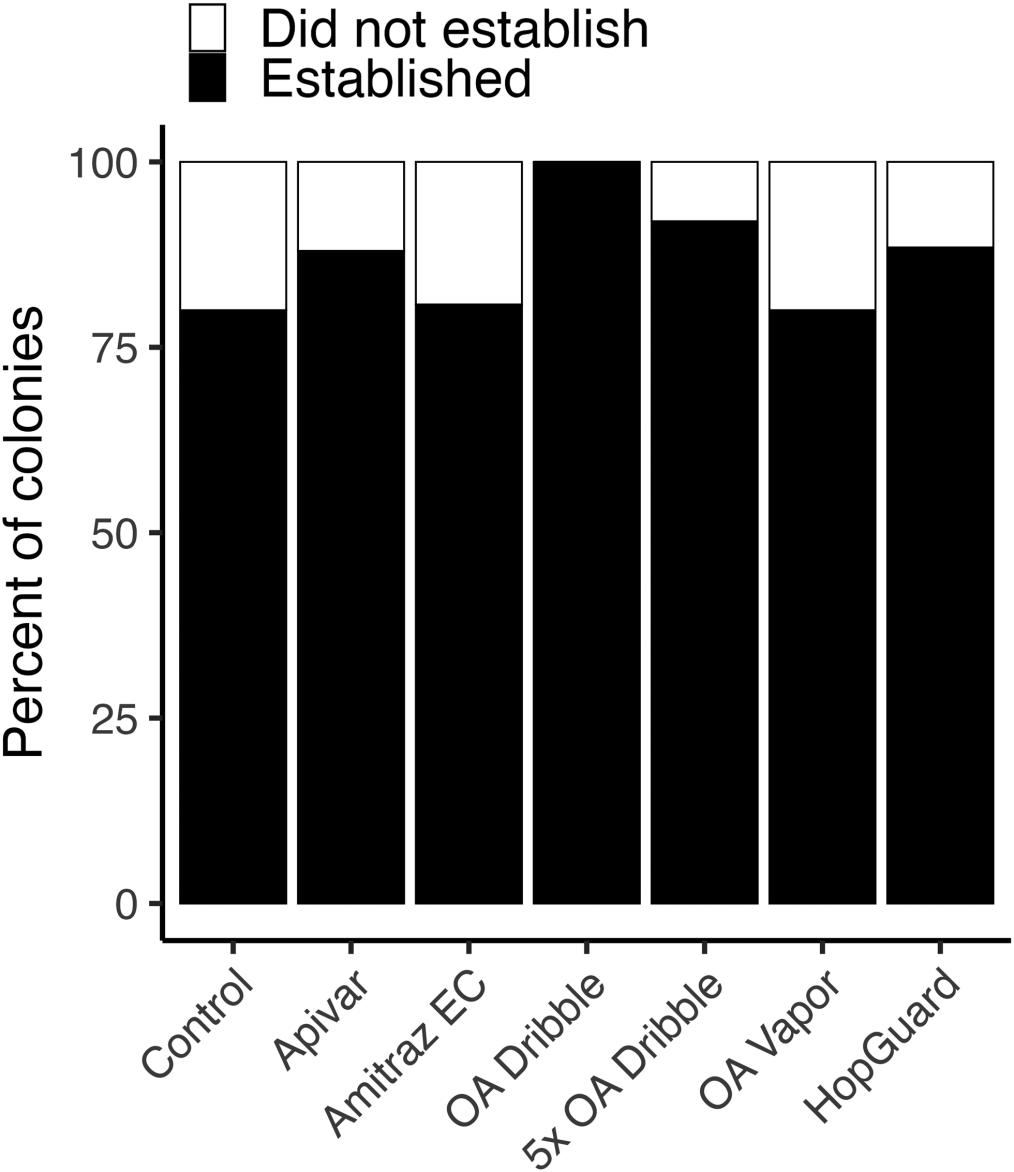
Percent of *Apis mellifera* colonies in which queens did or did not successfully establish (begin laying worker eggs) after receiving a queen cell (immature queen) and being subjected to different miticide treatments to manage *Varroa destructor* mites.

We did not observe evidence of treatment damage to colonies based on queen establishment, the occurrence of queen and colony issues, colony strength, or hive weight. Based on confirmed presence of worker brood, most queens (87.0%; 154/177) successfully established and began laying worker eggs (Fig. 3), with no statistically significant differences among groups (*F* = 1.24; df = 6, 170; *P *= 0.288). All treatments had at least as high an establishment rate as the Control group (80%). Queens which successfully established laid their first eggs at earliest on day 9 and at latest on day 28 (mean: day 14.5). This corresponds to the earliest queens beginning to lay eggs 7 days after we introduced queen cells. The prevalence of other issues (queenlessness, drone laying queens, EFB, and colony weakness) was relatively even across treatments (Supplementary Fig. S3).

**Fig. 4.**
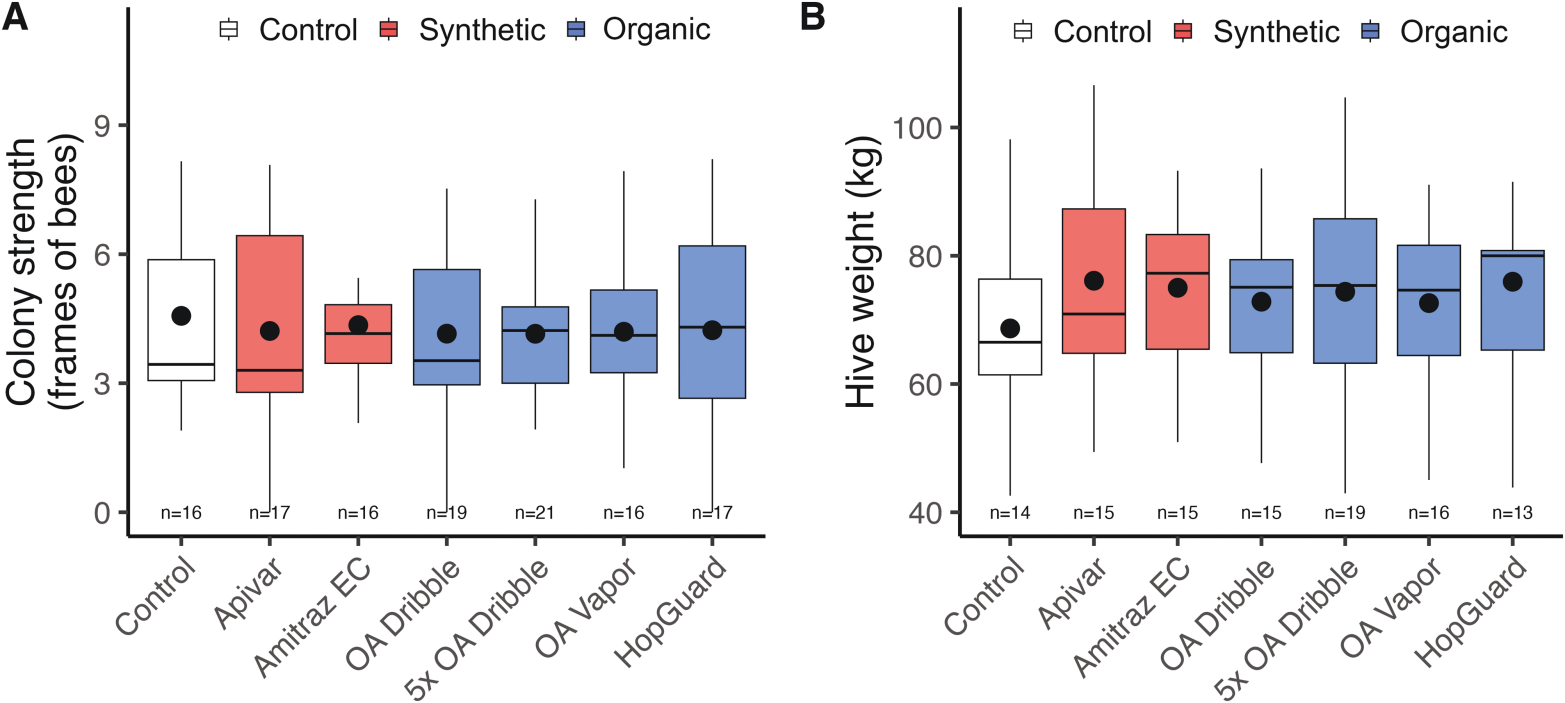
Colony strength and productivity metrics of *Apis mellifera* colonies that received different miticides against *Varroa destructor* mites after receiving a queen cell (immature queen). A) Colony strength 77 d after splitting. B) Hive weight at honey harvest (including supers; corrected for differing numbers of supers). In both panes, the boxplots show directly plotted data including median (horizontal line) interquartile range (body of box), and range (whiskers), while the points represent the model-predicted mean. There was no significant treatment effect on either metric.

There was no detectable impact of treatment on colony strength on day 77 (*F* = 0.0784; df = 6, 121; *P *= 0.998; Fig. 4A). Following honey production, all treatments had numerically heavier hives than the untreated Control group, (Fig. 4B), though the differences in hive weight between treatments were not statistically significant (χ^2^ = 3.02; df = 6; *P *= 0.807). In an exploratory analysis, we estimated that colonies treated with an effective miticide (Amitraz EC, HopGuard, 5× OA dribble, Apivar, OA dribble) were 4.03 kg heavier (−1.82, 9.80; 95% CI) than Control and OA vapor treated colonies, though this was not statistically significant (χ^2^ = 1.83, df = 1, *P *= 0.176).

## Discussion

To encourage broad adoption of IPM practices against *Varroa*, it is critical to develop methods that fit in the current production system of large-scale beekeepers. In this study, we showed that miticides based on oxalic acid and hops beta acids can be effectively integrated with a widely used colony increase practice: providing new splits with a queen cell. This combination of cultural control and organic chemical control could be implemented in many commercially managed colonies, and this study provided information to inform the choice of treatments. For different beekeepers, different considerations would carry different weight. However, among the organic treatments tested, the oxalic acid dribble 18 d after splitting appeared to balance important considerations especially well: high efficacy, safety to colonies, efficiency of application, and low cost.

Only a few previous studies have compared OA vapor and OA dribble head-to-head, with mixed results (Al Toufailia et al. 2015, Gregorc et al. 2016). While we did not find a statistically significant difference in efficacy between the dribble and vapor application methods of oxalic acid (OA), the drastic difference in estimated efficacy (75.5% for dribble and 44.3% for OA vapor)—and the result that OA vapor was the only treatment not statistically different from the negative control—suggest that the dribble method is preferable under these methods and conditions. Our conclusion stands in contrast to that of Al Toufailia et al. (2015) who tested multiple dosages of OA vapor and OA dribble in broodless colonies in winter and concluded that vapor was a preferable mode of application as it achieved the same level of control at lower doses of OA than the dribble application. Like Al Toufailia et al. (2015), we obtained high efficacy against *Varroa* with comparable applications (3.2% oxalic acid applications in our study and in their highest tested dose). However, we obtained contrasting results for the vapor method even though we applied it to hives of similar volume (42.5 L in the present study; 56.4 L in theirs). They obtained high efficacy (81%) with a 0.56-g dose of pure oxalic acid, and very high efficacy (97% and 93%) with 1.125- and 2.25-g doses (Al Toufailia et al. 2015). In contrast, the estimated efficacy of 44% we obtained with a 1-g dose of oxalic acid dihydrate vapor (equivalent to 0.71 g pure oxalic acid) was much lower than we anticipated. This might have to do with the season (spring vs. winter), ambient temperatures, the chemical form of the acid, or perhaps specifics of the applicator device. Al Toufailia et al. (2015) reported efficacy based on measured *Varroa* drop during a test treatment and a contrast treatment, while our study used measurements of *Varroa* infestation before and after treatment to calculate efficacy. These differences in methods may influence the comparability of results, though the implications of the methodological difference are not completely clear. Since a 1-g dose was inferior to higher doses when colonies contained ample brood (Jack et al. 2021), and small amounts of capped brood would have been present during treatment in many of our colonies, it is also possible that the presence of small amounts of brood may interfere more with *Varroa* control from vapor applications than from dribble applications. More work is needed to tease apart the multiple potential factors at play, given the interest in optimizing different kinds of oxalic acid application (Jack et al. 2020). We obtained marginally higher estimated efficacy with the multiple dribble application than with the single application, but we do not think the estimated increase in efficacy justifies the additional apiary visits for application. Regarding the efficacy of HopGuard, our results align with those of DeGrandi-Hoffman et al. (2014) who found excellent short-term control of *Varroa* infestations. In our study, the HopGuard treatment had the highest estimated efficacy among organic treatments on day 77, so this could be an attractive choice when high efficacy is given more weight than other factors such as treatment cost. Observing increases in *Varroa* infestations between day 77 and honey harvest is expected as there were no treatments in place, and because the bee population in colonies was more stable in the honey production period, compared with the rapid increases from colony establishment to the day 77 checks.

From the standpoint of safety to colonies, no miticide treatments appeared to damage queens, colonies, or colony productivity. This was even though we placed strip formulations (Apivar and HopGuard) adjacent to queen cells. Similarly, five sequential applications of OA dribble seemed to cause no issues during the experiment, which aligns with other studies showing no effects of OA on colonies at these doses (reviewed in Rademacher and Harz 2006), but are in contrast to others that have found negative effects (Higes et al. 1999, Schneider et al. 2012). As other workers have noted (Jack et al. 2021), the effects of repeated exposure would be worth testing in colonies exposed long-term. Nevertheless, the lack of short-term negative impacts from five sequential oxalic acid applications further indicates that the use of a single oxalic acid dribble should be considered low-risk, at least in the spring season.

From the standpoint of efficiency of application and cost, OA dribble, OA vapor, and HopGuard were all efficient in that they only required intervention on the day the queen cell was placed and 18 d after splitting. For beekeepers who are already splitting their colonies and installing queen cells, the time required to add treatment to this protocol would be ~2 to 3 min. If we assume that the most effective treatment timing coincides with the lowest brood area, the optimal date to treat would be somewhat variable as it depends on the timing of queen mating and egg-laying which vary among colonies and cohorts of colonies. Regarding cost of materials for treatments, the OA dribble and vapor applications are inexpensive (~$0.15 per application; price from Mann Lake Ltd., Hackensack, MN, USA), whereas HopGuard is more expensive (~$4.50 per colony for 2 strips; price from Mann Lake Ltd.). *Varroa* treatments can be an important portion of annual colony management expenses (DeGrandi-Hoffman et al. 2019) making cost relevant to beekeepers’ treatment decisions. It would be useful to conduct cost-benefit analyses of the choices between using mated queens and using queen cells, and of treatment choices at specific opportunities. When expanding on such analyses, it is important to keep in mind that different beekeeping operations have specific needs (eg for strong colonies at certain times of year) and have different constraints (eg labor availability at certain times of year).

In conclusion, our results strongly support the ability of organic miticides to effectively control *Varroa* mites when they are strategically applied to coincide with the temporary minimum of capped brood area which occurs ~18 d after splitting colonies (when using queen cells). The estimated efficacies in this study are all relative to negative control colonies that received the cultural control (splitting and providing a queen cell) but no chemical treatment. Therefore, in comparison to colonies that are split and receive a mated queen—or colonies that are not split at all—the efficacy against *Varroa* would be higher. Many beekeepers in the United States start colonies by splitting and providing queen cells, but it seems that few beekeepers use this opportunity to manage *Varroa*. Oxalic acid dribble applied 18 d after splitting, in particular, balanced several important considerations through high efficacy control of *Varroa*, no harm to colonies, low cost, and low labor to apply.

## Supplementary material

Supplementary material is available at *Journal of Economic Entomology* online.

toaf126_suppl_Supplementary_Material

## Data Availability

Data and code are available at https://doi.org/10.5281/zenodo.15733144.
