# Supplementary material for: Treating newly split Apis mellifera honey bee colonies with organic miticides—an opportunity for Integrated Pest Management of Varroa destructor mites (Mesostigmata: Varroidae)
Source: J Econ Entomol. 2025 Jul 1;118(4):1495–503. doi: 10.1093/jee/toaf126 (PMC12412293; doi:10.1093/jee/toaf126)
Supplement: toaf126_suppl_Supplementary_Material [file toaf126_suppl_supplementary_material.docx]

**Supplementary Material**

**Article Title:** Treating newly split Apis mellifera honey bee colonies with organic miticides – An opportunity for Integrated Pest Management of Varroa destructor mites (Mesostigmata: Varroidae)

**Journal:** *Journal of Economic Entomology*

**Authors:** Dan Aurell^1^*, Selina Bruckner^1^, Todd D. Steury^2^, Geoffrey R. Williams^1^*

**Author Affiliations:** ^1^Department of Entomology and Plant Pathology, Auburn University, Auburn, AL 36849, USA; ^2^College of Forestry, Wildlife and Environment, Auburn University, Auburn, AL 36849, USA; * Corresponding authors; emails: [sda0022@auburn.edu](mailto:sda0022@auburn.edu) (DA), [grw0010@auburn.edu](mailto:grw0010@auburn.edu) (GRW)

**Supp. Fig. S1.** Experiment timeline. The timing of actions, assessments, and treatments are shown relative to the experiment day. Four cohorts of experimental colonies were started on four different dates, as indicated, and all treatments were represented in each cohort. The initial number of splits in each treatment group is shown. When producing queen cells, larvae were grafted 10 days before the queen cells were installed in new splits.

**
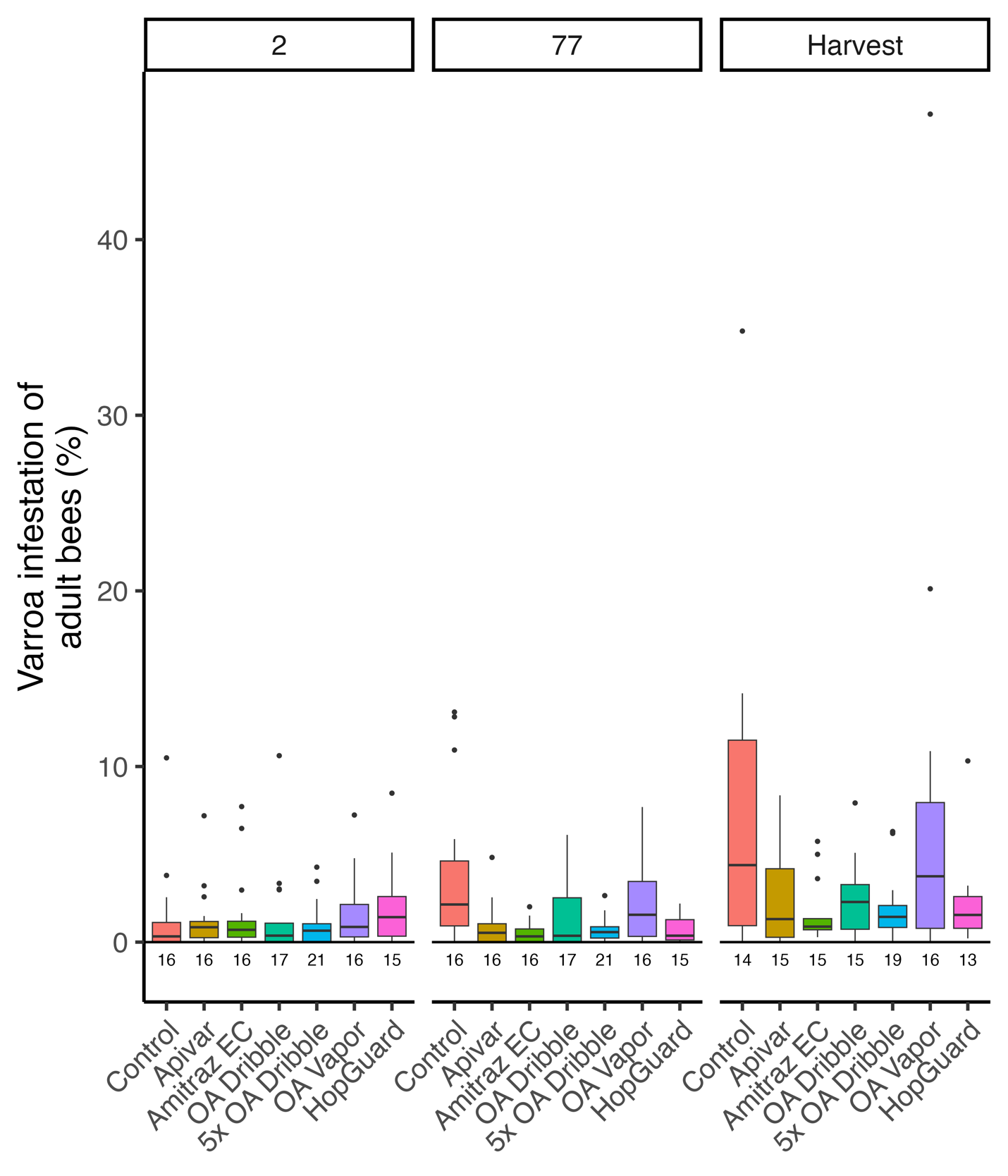
Supp. Fig. S2.** *Varroa destructor* infestations (on day 2, 77, and at honey harvest) of *Apis mellifera* honey bee colonies that received a queen cell (immature queen) and were subjected to different miticide treatments. The ends of the box indicate the upper and lower quartiles, the horizontal line indicates the median, the vertical lines indicate the range of the data, with outliers identified as points if they exceed the upper quartile by more than 1.5 times the interquartile range.


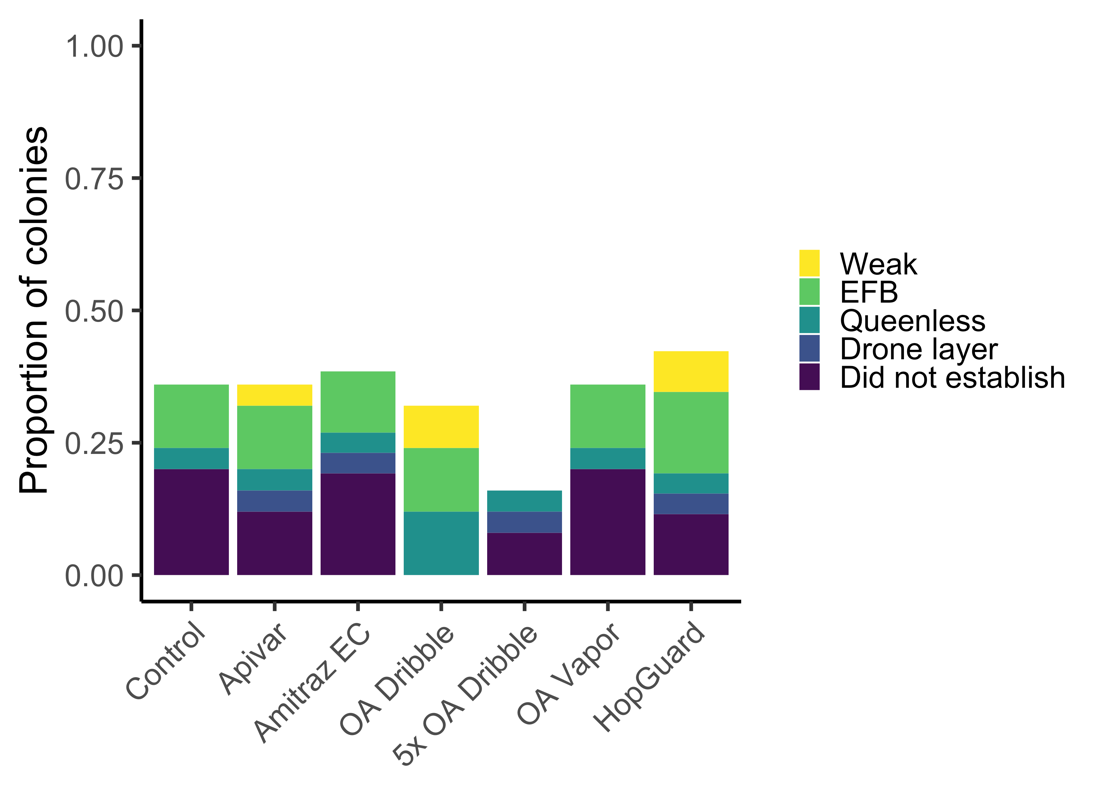


**Supp. Fig. S3.** The prevalence of issues other than *Varroa destructor* infestations in *Apis mellifera* honey bee colonies that received a queen cell (immature queen) and were subjected to different miticide treatments.

**Supp. Table S1.** Linear and linearized models used to analyze *Varroa destructor* infestation rates of *Apis mellifera* honey bee colonies, the success of queen establishment, the strength of
*A. mellifera* colonies, and weight of hives at honey harvest. Models were fitted using the *MASS* and *lme4* packages in R 4.2.2.

| No. | Response variable description | Model formula* | Model type |
| --- | --- | --- | --- |
| 1 | Number of *Varroa* in sample of adult bees on day 77 (integer) | nVarroa ~ Treatment + preVarroa + offset(log(nBees)) | GLM, Neg. binomial  (log link) |
| 2 | Number of *Varroa* in sample of adult bees at honey harvest (integer) | nVarroa ~ Treatment + preVarroa + Days + offset(log(nBees)) | GLM, Neg. binomial  (log link) |
| 3 | Success of queen establishment (binary) | Established ~ Treatment | GLM, Binomial (logit link) |
| 4 | Frames of adult bees on day 77 (continuous) | Frames ~ Treatment | LM, Gaussian |
| 5 | Weight of hives at honey harvest (continuous) | Weight ~ Treatment | LMM, Gaussian |
| 6 | Number of *Varroa* in sample of adult bees | nVarroa ~ Treatment + TimePoint + Treatment:TimePoint +  random(1\|ColonyID) +  offset(log(nBees)) | GLMM,  Neg. binomial  (log link) |

preVarroa = “percent *Varroa* infestation rate of adult bees before treatment” (continuous); nBees = “number of bees in sample” (integer); Days = “number of days elapsed from splitting to honey harvest” (integer). *Model formulas are written in the form required by the *MASS* and *lme4* packages (Venables et al., 2002; Bates et al., 2015).

**Supp. Table S2.** Model-predicted *Varroa* infestation rates and confidence intervals in new *Apis mellifera* honey bee colonies started with queen cells (immature queens) and subjected to treatments with natural and synthetic miticides. Predictions for day 77 and 105 are based on a set value of the covariate day 2 infestation rate. This was set to 1.42 to reflect the mean % *Varroa* infestation rate on day 2 for colonies that were included in the day 77 data set.

| Day | Treatment | n | % *Varroa* infestation [95% CI] |
| --- | --- | --- | --- |
| 2 | All treatments | 117 | 1.42 |
| 77 | Control | 16 | 3.83 [2.24, 6.56] |
| 77 | Apivar | 16 | 0.76 [0.41, 1.38] |
| 77 | Amitraz EC | 16 | 0.48 [0.25, 0.92] |
| 77 | OA Dribble | 17 | 0.94 [0.53, 1.66] |
| 77 | 5x OA Dribble | 21 | 0.68 [0.40, 1.17] |
| 77 | OA Vapor | 16 | 2.13 [1.23, 3.69] |
| 77 | HopGuard | 15 | 0.66 [0.35, 1.25] |
| 105 | Control | 14 | 6.04 [3.53, 10.35] |
| 105 | Apivar | 15 | 2.33 [1.36, 4.01] |
| 105 | Amitraz EC | 15 | 1.47 [0.84, 2.57] |
| 105 | OA Dribble | 15 | 2.17 [1.26, 3.73] |
| 105 | 5x OA Dribble | 19 | 1.92 [1.18, 3.14] |
| 105 | OA Vapor | 16 | 6.72 [4.07, 11.10] |
| 105 | HopGuard | 13 | 2.26 [1.26, 4.05] |

**Supp. Table S3.** Results of linear hypothesis tests of efficacy between treatments (all pairwise comparisons) against *Varroa destructor* applied to new *Apis mellifera* honey bee colonies started with queen cells (immature queens). Contrasts are made for efficacy on day 77. *P*-values are adjusted for multiple comparison (21 comparisons; single-step method) and *P*-values less than 0.05 are identified in bold font.

| Contrast | Z statistic | *P*-value |
| --- | --- | --- |
| Apivar - Control | -3.95 | **0.002** |
| Amitraz EC - Control | -4.83 | **< 0.001** |
| OA Dribble - Control | -3.52 | **0.008** |
| 5x OA Dribble - Control | -4.44 | **< 0.001** |
| OA Vapor - Control | -1.49 | 0.748 |
| HopGuard - Control | -4.15 | **0.001** |
| Amitraz EC - Apivar | -1.00 | 0.953 |
| OA Dribble - Apivar | 0.51 | 0.999 |
| 5x OA Dribble - Apivar | -0.24 | 1.000 |
| OA Vapor - Apivar | 2.50 | 0.158 |
| HopGuard - Apivar | -0.29 | 1.000 |
| OA Dribble - Amitraz EC | 1.52 | 0.732 |
| 5x OA Dribble - Amitraz EC | 0.82 | 0.983 |
| OA Vapor - Amitraz EC | 3.44 | **0.011** |
| HopGuard - Amitraz EC | 0.70 | 0.992 |
| 5x OA Dribble - OA Dribble | -0.79 | 0.986 |
| OA Vapor - OA Dribble | 2.04 | 0.391 |
| HopGuard - OA Dribble | -0.80 | 0.985 |
| OA Vapor - 5x OA Dribble | 2.89 | 0.059 |
| HopGuard - 5x OA Dribble | -0.07 | 1.000 |
| HopGuard - OA Vapor | -2.75 | 0.086 |

**Supp. Table S4.** Efficacy estimates on day 77 and 105 of natural and synthetic miticides against *Varroa destructor* applied to new *Apis mellifera* honey bee colonies started with queen cells (immature queens).

| Day | Treatment | n | Efficacy (%) [95% CI] |
| --- | --- | --- | --- |
| 77 | Apivar | 16 | 80.28 [55.70, 91.26] |
| 77 | Amitraz EC | 16 | 87.46 [70.79, 94.66] |
| 77 | OA Dribble | 17 | 75.50 [46.39, 88.88] |
| 77 | 5x OA Dribble | 21 | 82.16 [62.05, 91.79] |
| 77 | OA Vapor | 16 | 44.30 [-20.79, 74.33] |
| 77 | HopGuard | 15 | 82.67 [60.23, 92.45] |
| 105 | Apivar | 15 | 61.41 [16.65, 82.23] |
| 105 | Amitraz EC | 15 | 75.63 [46.44, 88.97] |
| 105 | OA Dribble | 15 | 64.09 [22.56, 83.45] |
| 105 | 5x OA Dribble | 19 | 68.15 [34.66, 84.82] |
| 105 | OA Vapor | 16 | -11.17 [-132.57, 47.43] |
| 105 | HopGuard | 13 | 62.60 [15.03, 83.43] |
